# Rapid preparation of rodent testicular cell suspensions and spermatogenic stages purification by flow cytometry using a novel blue-laser-excitable vital dye

**DOI:** 10.1016/j.mex.2014.10.002

**Published:** 2014-10-16

**Authors:** Rosana Rodríguez-Casuriaga, Federico F. Santiñaque, Gustavo A. Folle, Elisa Souza, Beatriz López-Carro, Adriana Geisinger

**Affiliations:** a Departamento de Biología Molecular, Instituto de Investigaciones Biológicas Clemente Estable (IIBCE), Montevideo, Uruguay; b Servicio de Citometría de Flujo y Clasificación Celular (SECIF), IIBCE, Montevideo, Uruguay; c Sección Bioquímica, Facultad de Ciencias, Universidad de la República, Uruguay

**Keywords:** Spermatogenesis, Mouse, Testis, Flow cytometry, Vybrant DyeCycle Green

## Abstract

Availability of purified or highly enriched fractions representing the various spermatogenic stages is a usual requirement to study mammalian spermatogenesis at the molecular level. Fast preparation of high quality testicular cell suspensions is crucial when flow cytometry (FCM) is chosen to accomplish the stage/s purification. Formerly, we reported a method to rapidly obtain good quality rodent testicular cell suspensions for FCM analysis and sorting. Using that method we could distinguish and purify early meiocytes (leptotene/zygotene stages, L/Z) from more advanced ones (pachytene, P) in guinea pig, which presents an unusually high content of early stages. Here we present an upgrade of that method with improvements that enabled the obtainment of high-purity meiotic substages also from mouse testis, namely:

• Shortening of the mechanical disaggregation time to optimize the integrity of the suspension.
• Elimination of the 25 μm-filtration step to ensure the presence of large P cells.
• Inclusion of a non-cytotoxic, DNA-specific, 488 nm-excitable vital fluorochrome (Vybrant DyeCycle Green [VDG], Invitrogen) instead of Hoechst 33342 (requires UV laser, which can damage nucleic acids) or propidium iodide (usually related to dead/damaged cells). As far as we know, this is the first report on the use of this fluorochrome for the discrimination and purification of meiotic prophase I substages.

## Graphical abstract

**Figure:**
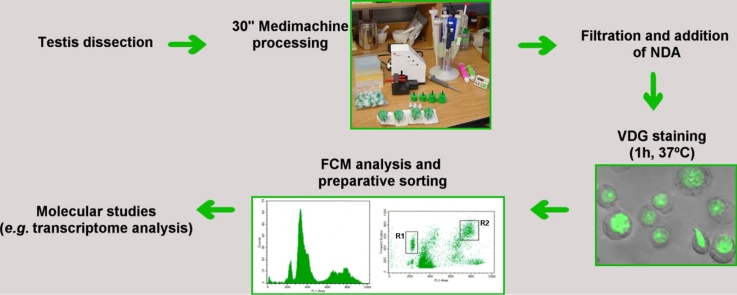

## Methods

### Spermatogenic stages purification by FACS using a blue-laser-excitable vital dye

Heterogeneity of mammalian testis is a major difficulty for the understanding of spermatogenesis bases, since pure or enriched cell populations representing the different stages of sperm development are required for most molecular analyses [1]. Diverse strategies such as Staput [2,3], centrifugal elutriation [1] and flow cytometry (FCM) [4–6] have been used to obtain enriched or purified testicular cell populations for subsequent differential gene expression studies. Cells must be in suspension for most enrichment/purification approaches. Ideally, the cell suspension should represent as much as possible the original tissue, lack cell clumps [1], and have a high proportion of viable cells as well as few multinucleates, which tend to form as a consequence of the syncytial nature of the seminiferous epithelium [7,8].

We are hereby presenting an upgrade of a previously reported method for the rapid preparation of rodent testicular cell suspensions [9,10]. The original protocol was used in combination with either the UV-excitable Hoechst 33342 vital dye, or with propidium iodide (PI). Although the conditions formerly used with PI (mechanical stress, high dye concentration and long exposure time) favored its entrance to the cells in the suspension, this fluorochrome would not be the first choice for preparative cell sorting as it is usually excluded from intact cells.

The optimization of the method with a blue-laser-excitable DNA-specific vital dye (Vybrant DyeCycle Green [VDG], Invitrogen) has had a direct effect on the integrity of the purified fractions, turning them more suitable for downstream expression profiling. Besides, the inclusion of VDG afforded very interesting cytometric profiles with a significant number of cell populations distinguishable in the dot plots, even in species with no peculiar abundancy of certain stages such as L/Z in mouse [11]. Within 2C cells, different subpopulations can also be distinguished, but their identities are yet to be determined.

## Method detail

### Animals

CD-1 Swiss mice were employed in this work. The age range analyzed was 10–27-day-old juveniles, and 40-day-old young adults. 5–7 specimens were used in each case, and analyzed individually to evaluate specimen variability and sorting consistency. All experimentation procedures were performed in accordance with the National Law of Animal Experimentation 18,611 (Uruguay).

### Preparation of the cell suspension

(1) Kill the specimen to be used following the recommendations of the specialized committees (in Uruguay, National Commission for Animal Experimentation [CNEA]; experimental protocol 001/02/2012). In our case, an overdose of pentobarbital was supplied, except for the youngest specimens in which cervical dislocation was performed.
(2) Dissect the testes following standard procedures, and place them on ice in a 60 mm glass Petri dish containing 5 mL of ice-cold DMEM supplemented with 10% fetal calf serum (ice-cold freshly prepared DPBS can be used instead, in this and subsequent steps).
(3) Remove the tunica albuginea and cut the decapsulated testes into pieces of 2–3 mm on each side.
(4) Process pieces in a Medimachine (BD Biosciences), an automated electro-mechanical solid-tissue disaggregator [9,10]. Tissue pieces are disaggregated inside a disposable grinder unit containing a perforated stainless-steel screen and a metal rotor. To do so, place 1 mL of cold supplemented DMEM and 4–5 of these pieces in the disposable unit, switch on the disaggregator, and process for 30 s.
(5) Recover the resulting cell suspension from the disaggregation unit using a 3–5 mL syringe without needle.
(6) Filter through a 50 μm nylon mesh previously soaked with 0.5 mL supplemented DMEM.
(7) To prevent cell clumping add NDA (2-naphthol-6,8-disulfonic acid, dipotassium salt) to a final concentration of 0.2%.
(8) Repeat step 6, and place on ice.
(9) Finally, take an aliquot to count in a Neubauer chamber and adjust concentration to 3–5 × 10^6^ cells/mL with supplemented DMEM or DPBS. At least 4–5 × 10^7^ cells/g of testis material are usually obtained. Viability can be checked by Trypan blue exclusion. To do so, mix 100 μL of cellular suspension, 200 μL of DPBS and 300 μL of 0.4% Trypan blue, and count in a Neubauer chamber. This method usually renders above 90% viability.

### Flow cytometric analysis

**Fig. 1:**
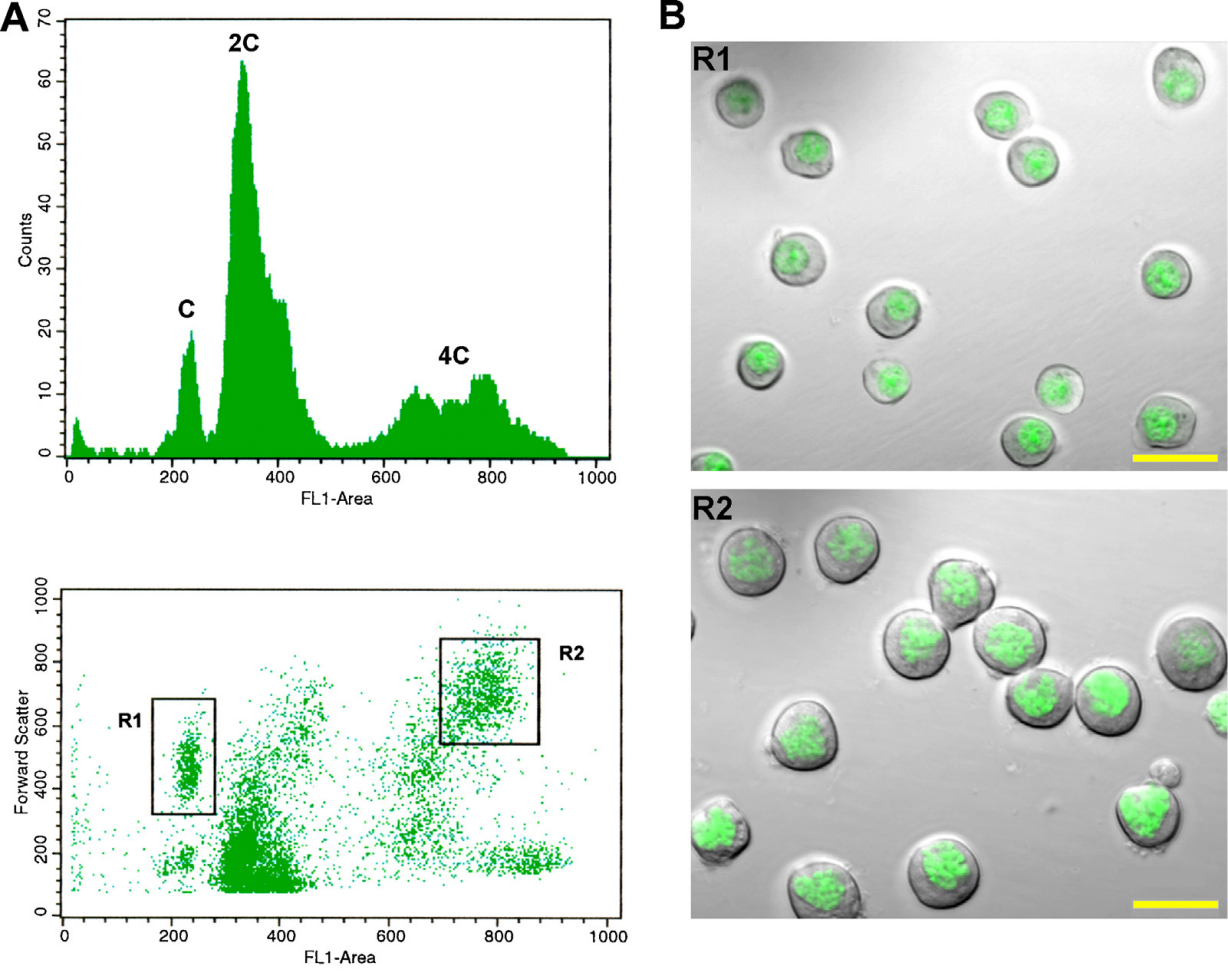
(A) Flow cytometric analysis of a 21-day-old mouse testicular cell suspension stained with VDG. A dot plot depicting forward scatter (FSC-H) vs. VDG fluorescence intensity and its corresponding histogram are shown. Peaks corresponding to cell populations differing in their DNA content (C, 2C and 4C) are indicated. Sorting gates chosen within the C and 4C populations are shown as well (R1 and R2, respectively). (B) Analysis of sorted cells from the indicated gates (C upper region [R1], and 4C right upper region [R2]). Sorted cells were analyzed under laser confocal (VDG green fluorescence) and differential interference contrast (DIC) microscopy. Note the homogeneity and integrity of the classified material. Bars correspond to 20 μm.

Any flow sorter equipped with a standard 488 nm blue laser can be used for the analysis and separation of cells stained with VDG (Invitrogen). We have used a FACSVantage (BD Biosciences) flow sorter equipped with a Coherent argon ion laser tuned to emit at 488 nm. VDG is a DNA-specific vital dye with non-cytotoxic reported effects that allows cytometric analysis based on DNA content using a blue laser as excitation source. Interestingly, it also renders differential chromatin staining patterns that resemble those obtained with Hoechst dyes under a fluorescence microscope (see Fig. 1).

(1) For VDG staining, add fluorochrome at a final concentration of 10 μM to the cell suspension, and incubate for 1 h at 37 °C in the dark.
(2) Laser power is set to 100 mW and a 530/30 band pass filter is used to collect VDG-emitted fluorescence in FL1.
(3) Perform FCM measurements with a 70 μm nozzle. Use CellQuest software (BD Biosciences) to analyze the following parameters: forward scatter (FSC-H); side scatter (SSC-H); total emitted fluorescence or pulse-area (FL1-A); and duration of fluorescence emission or pulse-width (FL1-W).
(4) For purification of testis cell populations representing different spermatogenic stages, set sorting mode in Normal-R or Normal-C, using 3 sorted drops as envelope. Keep sample and collection tubes at 3–4 °C by using a refrigeration unit. Adjust sample differential pressure to analyze cells at a rate of 500–1000 per second.
(5) Collect sorted cells into 12 mm × 75 mm polystyrene tubes containing 0.5 mL separation medium. If downstream RNA extraction is planned, work under RNAase-free conditions and collect cells into sterile tubes containing PBS treated with 0.1% DMPC (PBS-DMPC). Subsequently, spin down the cells (500 g, 10 min, 4 °C), wash with PBS-DMPC, freeze in liquid nitrogen and store at −80 °C. Alternatively, cells can be directly classified onto plastic Petri dishes using Clone-Cyt software (BD), and analyzed under laser confocal and differential interference contrast (DIC) microscopy to determine cellular integrity and homogeneity of sorted fractions. Moreover, identity of classified cells can be determined by immunocytochemical assays using cell type-specific antibodies. In this work, immunodetection of a meiotic cell-specific protein was performed essentially as described before [11].

By this method we have classified different stages of mouse spermatogenesis based on DNA content, cellular size and granularity (complexity). Some representative results are illustrated in the figures. Fig. 1 shows purified round spermatids (C DNA content) and primary spermatocytes (4C DNA content) obtained through the sorting gates indicated in the dot plot. As can be seen, sorted cells preserve their cytoplasms and exhibit overall conserved morphology, which is critical when the method is employed with preparative aims (*e.g.* downstream RNA extraction for gene expression profiling).

**Fig. 2:**
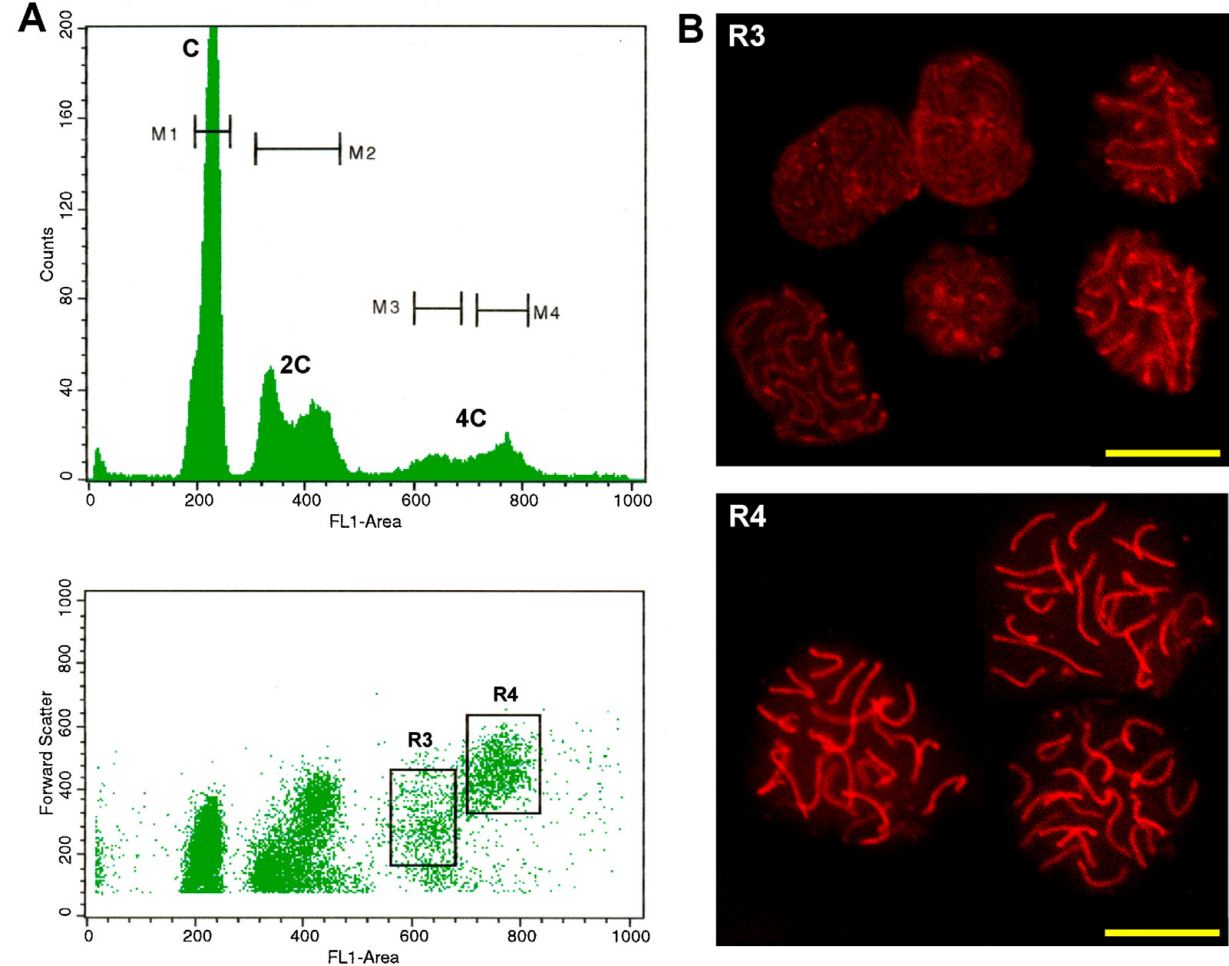
(A) Flow cytometric profiles obtained from the analysis of a 25-day-old mouse testicular cell suspension. A FSC-H vs. VDG fluorescence intensity dot plot and its corresponding histogram are shown. Peaks pertaining to C, 2C and 4C cell populations are indicated. Sorting gates within the 4C population are indicated (R3 and R4). (B) Analysis of sorted cells from R3 and R4 regions. Laser confocal microscopy of immunodetections using an antibody (Acris) raised against the Ct region of mouse Sycp3 (synaptonemal complex [SC] protein 3, a lateral element component) is shown. Immunodetections were performed basically as described in [11], except that a Texas Red-tagged goat anti-rabbit secondary antibody (Abcam) was employed. As can be seen, cells coming from R3 region correspond to early stages of the first meiotic prophase (L and Z) in which simple axes (L) and short stretches of SCs (Z) are present, while those classified from R4 region are mid-to-late primary spermatocytes (P stage) with completely assembled SCs. Bars correspond to 10 μm.

More refined classifications can be also achieved through this method. In a previous work we succeeded in purifying early and late spermatocytes using guinea pig as experimental model and long exposure to PI [11], but we were not able to reproduce this purification technique for mouse testis, which has a comparatively scarse representation of early (L/Z) spermatocytes. The upgrade presented here – especially the incorporation of VDG – has enabled the purification of high-purity meiotic substages also from mouse testis (Fig. 2). Good quality RNA has been obtained from the purified fractions, and used in transcriptomic studies along mouse first meiotic prophase (manuscript in preparation).

The simplicity, brevity and reproducibility of the presented protocol (it is totally operator-independent), combined with the good quality of the resulting testicular cell suspensions (viability levels >90%; multinucleates ≤8%) and classified fractions (preservation of cytoplasms, RNA integrity), turn this method an interesting choice for FCM analysis and sorting using VDG vital dye and a standard blue laser as excitation source.
